# Anticoagulation Management During ECMO: Narrative Review

**DOI:** 10.1016/j.jhlto.2025.100216

**Published:** 2025-01-20

**Authors:** Jaromir Vajter, Oksana Volod

**Affiliations:** a Department of Anaesthesiology, Resuscitation, and Intensive Care Medicine, 2nd Faculty of Medicine, Charles University and Motol University Hospital, Czech Republic; b Department of Pathology and Laboratory Medicine, Cedars Sinai Medical Center, Los Angeles, CA

**Keywords:** ECMO, Anticoagulation, UFH, LMWH, Argatroban, Bivalirudin

## Abstract

Extracorporeal membrane oxygenation (ECMO) is a critical intervention for patients with severe respiratory or cardiac failure, requiring careful management of anticoagulation to prevent thromboembolic complications. This review examines current practices and challenges in ECMO anticoagulation, focusing on strategies, agents, and emerging insights. Unfractionated heparin (UFH) remains the most commonly used anticoagulant, monitored via activated partial thromboplastin time (aPTT) or activated clotting time (ACT). Increasing attention is given to alternative tools like anti-Xa and viscoelastic assays (VEA), which offer potentially more reliable results. Supplementation with antithrombin should be considered if levels fall below 50%–70% to optimize heparin efficacy. Low molecular weight heparin (LMWH) is occasionally used due to its predictable pharmacokinetics, though challenges in dosing and reversal limit its application. Direct thrombin inhibitors, such as bivalirudin, are valuable alternatives, particularly for patients with heparin-induced thrombocytopenia (HIT), though their cost and availability remain barriers. Anticoagulation in ECMO patients is complex, balancing the risks of thrombosis and bleeding. Factors such as patient age, underlying conditions, and ECMO-induced coagulopathies complicate management. Personalized anticoagulation protocols and point-of-care VEA are emerging as effective tools for improving therapy. Routine no-anticoagulation strategies are not recommended unless there are significant bleeding complications. Ongoing research into novel anticoagulants and the long-term impact of anticoagulation on ECMO outcomes is critical. Anticoagulation management in ECMO continues to evolve, focusing on individualized approaches, improved monitoring, and better outcomes. Standardized protocols and further research are essential for optimizing care in this high-risk population.

## Background

Extracorporeal membrane oxygenation is a life-saving intervention for critically ill patients with severe respiratory or cardiac failure. It supports gas exchange and hemodynamics by circulating blood through an external circuit. A key challenge in ECMO management is the prevention of thromboembolic complications, which require precise anticoagulation. UFH remains the most commonly used anticoagulant due to its rapid onset, ease of reversibility, and established monitoring protocols.^1^, ^2^ Additionally, LMWH is employed for its stable pharmacokinetics, though its use is limited by challenges in dose adjustment and reversal, particularly in high-bleeding risk scenarios.^3^ However, alternatives, such as direct thrombin inhibitors (DTI),^1^, ^2^, ^4^ are increasingly used in cases of heparin-induced thrombocytopenia,^1^, ^2^ offering more predictable effects with less variability.

Effective anticoagulation management requires rigorous biological monitoring to balance the risks of thrombosis and bleeding. Traditional tests like aPTT and ACT are widely used to monitor the anticoagulant effect of UFH. Still, both tests have limitations regarding variability and reliability in ECMO patients.^1^, ^2^, ^5^, ^6^, ^7^ Increasingly, anti-Xa levels are used to directly measure heparin activity, offering more precision in dosing adjustments. Moreover, VEA, such as rotational thromboelastometry (ROTEM) and thromboelastography (TEG), provide a more comprehensive assessment of clot formation and stability, allowing for real-time adjustments in anticoagulation and blood product management.^8^, ^9^, ^10^

This review will explore the most commonly used anticoagulants in ECMO therapy and highlight the evolving approaches to monitoring anticoagulation efficacy and safety, aiming to provide a detailed understanding of current practices and future directions in ECMO anticoagulation management.

## Methodology

This review article aims to comprehensively analyze anticoagulation management during adult ECMO support and is structured into three chapters. Each chapter utilizes a systematic and methodical approach to ensure an in-depth exploration of current practices, evidence, and personal insights into ECMO anticoagulation.

### Part 1: Analysis of guidelines and consensus statements

The first chapter is dedicated to reviewing and comparing major international societies' guidelines and consensus statements published between 2018 and 2024, focusing on ECMO anticoagulation. This includes the Society of Cardiovascular Anesthesiologists (SCA, 2021) Consensus Statement,^11^ the International Society on Thrombosis and Hemostasis (ISTH, 2023) Guidelines,^2^ the Extracorporeal Life Support Organization (ELSO, 2021) Guidelines,^1^ the American Association for Thoracic Surgery (AATS, 2022) Expert Consensus,^7^ the European Association for Cardio-Thoracic Surgery (EACTS)/ELSO/ The Society of Thoracic Surgeons (STS)/AATS (2020) Consensus,^6^ the International Society for Heart and Lung Transplantation (ISHLT, 2024) Consensus statement^12^ and The ISHLT/Heart Failure Society of America Guideline on Acute Mechanical Circulatory Support.^13^

This chapter aims to critically assess the differences and commonalities between these guidelines regarding anticoagulation strategies, dosing, monitoring parameters, and clinical decision-making frameworks.

### Part 2: Review of peer-reviewed studies and literature

The second chapter analyzed peer-reviewed clinical studies addressing anticoagulation in ECMO-supported patients. A systematic search of databases such as PubMed, Scopus, and Cochrane Library was conducted using the keywords "retrospective studies", "prospective studies", "randomized controlled trials", “ECMO”, “anticoagulation”, “unfractionated heparin ”, “direct thrombin inhibitors ”, “low molecular weight heparin ”, “viscoelastic assays”, and “thrombosis prevention”. Only studies published as full text in English in peer-reviewed journals between 2018 and 2024 and focused on adult patients undergoing ECMO for cardiopulmonary support were included.

The selected studies were evaluated based on their methodological rigor, sample size, clinical outcomes, and the relevance of anticoagulation strategies, including dosing regimens and monitoring tools (e.g., ACT, aPTT, anti-Xa, and VEA). A comparative analysis of study findings will be provided, highlighting best practices, efficacy, and safety of various anticoagulation protocols in ECMO.

### Part 3: Advances in anticoagulation monitoring and therapy in ECMO

The final chapter presents a personal perspective on the practical challenges and clinical decision-making processes in managing anticoagulation during ECMO support. Drawing from extensive clinical experience in critical care and thoracic anesthesia, this chapter will discuss real-world applications of anticoagulation strategies, including managing complex cases, balancing bleeding and thrombosis risks, and the role of personalized medicine. Insights into the limitations of current guidelines and how they apply to various patient populations (e.g., those with heparin resistance, HIT, or coagulopathy) will also be explored.

This methodology ensures a balanced and thorough review, combining guideline analysis, literature review, and practical experience to offer a holistic perspective on ECMO anticoagulation management.

## Analysis of guidelines and consensus statements

The practice of ECMO anticoagulation varies significantly across institutions. After analyzing the guidelines and consensus statements provided by major international societies, we found that there still needs to be a clear consensus highlighting one anticoagulant as superior to others or identifying a single laboratory method as the most accurate. Although several guidelines and consensus statements from international societies summarizing knowledge on ECMO support are available, only one society, ISTH, provides recommended dosing for individual anticoagulants such as UFH, Argatroban, and Bivalirudin.^2^ Other societies only present recommended monitoring methods and, in some cases, their reference ranges, such as aPTT, ACT, anti-Xa, aPTT ratio, and viscoelastic methods.^1^, ^5^, ^6^, ^7^, ^13^ The parameters, reference ranges, and anticoagulant doses are summarized in Table 1, Table 2. The 2020 EACTS/ELSO/STS/AATS expert consensus on post-cardiotomy extracorporeal life support in adult patients recommends that a TEG-driven algorithm should be considered for anticoagulation management (Class IIa, Level C recommendation), while the general principle of such an algorithm has been recommended, no precise algorithm have been specified.^6^ However, no international society has provided detailed guidance on the use of viscoelastic methods for monitoring and managing anticoagulation during ECMO. UFH anticoagulation during ECMO is typically monitored by targeting anti-Xa levels of 0.3–0.7 U/mL, an aPTT of 50–90 s or an ACT of 180–220 s. Studies have shown that using a lower-dose heparin regimen, targeting an ACT of 140–160 s, is associated with fewer major bleeding incidents compared to the therapeutic-dose heparin regimen, with no corresponding increase in thrombotic events. Anti-Xa monitoring has been found to correlate better with UFH dosing than aPTT, ACT, or aPTT ratio. Complications such as intracranial hemorrhage or gastrointestinal bleeding do not differ significantly between low and high UFH doses, and anti-Xa-based strategies have shown fewer bleeding events and lower mortality without an increase in thrombotic events.^14^, ^15^ Physicians must consider factors such as hemolysis, hyperbilirubinemia, and hyperlipidemia that may interfere with coagulation assays. In cases of heparin resistance or HIT, switching to DTIs like Argatroban or Bivalirudin is recommended, as these drugs act independently of antithrombin. Argatroban dosing should follow institutional protocols, starting at 0.2 μg/kg/min for patients with normal organ function and reduced doses for those with liver dysfunction, with aPTT-based monitoring recommended. DTIs can also be monitored using Ecarin Clotting Time (ECT), which is particularly useful for Bivalirudin and Argatroban. Routine no-anticoagulation strategies are not recommended in ECMO unless there are significant bleeding complications. Finally, ACT is influenced by clinical factors such as thrombocytopenia, platelet dysfunction, and elevated D-dimers and may not solely represent the effect of UFH. Anti-Xa assays may yield falsely low results in plasma-free hemoglobin levels above 50 mg/dL, triglycerides over 500 mg/dL, or bilirubin levels exceeding 6 mg/dL.^1^, ^5^, ^6^, ^7^, ^13^ According to a recently published ISHLT consensus statement, it is reasonable to consider a low-dose heparin protocol for VA ECMO during lung transplantation if adequate blood flow can be maintained.^12^

**Table 1:** Suggested Laboratory Levels During Anticoagulation with UFH for ECMO Anticoagulation^1^, ^2^, ^6^, ^7^, ^11^, ^12^, ^13^

Parameter	ISTH	ELSO	EACTS/ELSO/STS/AATS	AATS	ISHLT	SCA
ACT	180–220 s	180–220 s	160–220 s	180–220 s	180–220 s	180–220 s
APTT	50–70 s	60–90 s	50–80 s	N/A	50–70 s	60–80 s
anti-Xa activity	0.3–0.5 IU/mL	0.3–0.7 IU/mL	N/A	0.3–0.7 IU/mL	0.3–0.7 IU/mL	0.3–0.7 IU/mL
APTTr	2–2.5				1.5−2.5	
VEA	N/A	Monitor clot stability	A TEG-driven anticoagulation algorithm should be considered	N/A	Concurrent TEG and aPTT may cause excess anticoagulation	N/A
AT III monitoring and supplementation	Monitor in patients with thrombosis	More evidence needed	Monitor to detect HR	N/A	Monitoring recommended, loading dose 45 IU/kg	N/A

Abbreviations: aPTT: Activated Partial Thromboplastin Time; Anti-Xa Activity: Anti-Factor Xa Activity; aPTTr x times above the normal level of the institutional standard for therapeutic range aPTT; ATIII: antithrombin III; VEA: Viscoelastic Assays; ISTH: International Society on Thrombosis and Hemostasis; ELSO: Extracorporeal Life Support Organization; EACTS: European Association for Cardio-Thoracic Surgery; AATS: American Association for Thoracic Surgery; ISHLT: International Society for Heart and Lung Transplantation; SCA: Society of Cardiovascular Anesthesiologist; N/A: not available; HR: heparin resistance

**Table 2:** Anticoagulant Dosing based on ISTH^2^

Anticoagulant	Dose	Maintain
UFH	Bolus 50 – 100IU/kg	Continual infusion to achieve anticoagulation targets
Bivalirudin	0.02 – 0.05 μg/kg/min	Continual infusion to achieve anticoagulation targets
Argatroban	0.2 – 0.5 μg/kg/min	Continual infusion to achieve anticoagulation targets

Abbreviations: UFH: Unfractionated Heparin; IU: International Unit; mcg: microgram; kg: kilogramme; min: minute

## Review of peer-reviewed studies and literature

### Anticoagulation monitoring

#### Overview of aPTT Monitoring for UFH and DTIs in ECMO

##### aPTT monitoring for UFH

aPTT is commonly used to monitor anticoagulation with UFH during ECMO. Typical target ranges between 40 and 80 s, depending on institutional protocols. The mean and median values are typically around 60 s. The most frequently cited target is 60 s in clinical practice. Advantages include being readily available in most settings. It provides a broad assessment of the intrinsic coagulation pathway, which helps identify coagulation abnormalities and monitor anticoagulation. Limitations include the fact that aPTT results can be affected by factors such as fibrinogen levels, factor deficiencies, or lupus anticoagulants. Variability in reagents and instruments may lead to inconsistent results across different laboratories.^4^, ^16^, ^17^, ^18^, ^19^, ^20^, ^21^, ^22^, ^23^, ^24^, ^25^, ^26^, ^27^, ^28^

##### aPTT monitoring for DTIs

When using direct thrombin inhibitors such as argatroban and bivalirudin during ECMO, aPTT remains a crucial tool for anticoagulation monitoring, but the approach differs slightly due to the pharmacological properties of these agents.

##### Argatroban

Most studies recommend maintaining aPTT between 50–70 s, corresponding to 1.5–2.5 times the baseline aPTT. Variability depends on institutional thresholds and the risk of bleeding. Argatroban is hepatically metabolized, making it a preferred option in patients with renal dysfunction. Its effects on aPTT are dose-dependent and reversible upon cessation of the infusion. Prolongation of aPTT may present challenges, as it canbe disproportionately affected in patients with underlying coagulopathy or liver dysfunction.

##### Bivalirudin

Commonly cited targets are 50–75 s, or approximately 1.5–2.5 times the baseline aPTT. Some protocols prefer using anti-IIa activity for more precise monitoring when available. Bivalirudin is renally cleared and has a short half-life, providing predictable and adjustable anticoagulation. It demonstrates reduced interaction with other blood proteins compared to argatroban, which may provide more stable aPTT values. Renal dysfunction can prolong its half-life, which requires dose adjustments and closer monitoring.^19^, ^28^, ^29^

##### Conclusion

aPTT remains a vital monitoring tool for both UFH and DTIs in ECMO patients. While target ranges vary slightly between UFH, argatroban, and bivalirudin, understanding their pharmacokinetic properties and the influence of patient-specific factors is crucial for optimizing anticoagulation management. The summary of aPTT ranges for practical use is presented in Table 3.^16^, ^19^, ^20^, ^21^, ^22^, ^23^, ^24^, ^26^, ^27^

**Table 3:** Summary of aPTT Ranges for Practical Use^16^, ^19^, ^20^, ^21^, ^22^, ^23^, ^24^, ^26^, ^28^, ^37^

Anticoagulant	Target aPTT (Seconds)	Comments
UFH	40–80	Median ∼60; most common target in ECMO.
Argatroban	50–70	Adjusted for liver function; monitoring variability possible.
Bivalirudin	50–75	Preferred for stable dosing; adjust for renal function.

Abbreviations:UFH: Unfractionated heparin; ECMO: Extracorporeal Membrane oxygenation

#### Role of anti-Xa in anticoagulation monitoring during ECMO

The most commonly reported anti-Xa values for UFH range between 0.2–0.35 IU/mL, aligning with typical clinical practice for anticoagulation management during ECMO.^21^, ^28^, ^30^ Maintaining anti-Xa levels within this range provides adequate anticoagulation while minimizing the risk of bleeding or thrombotic complications. The suggested anti-Xa target for enoxaparin is 0.4–0.6 IU/mL, indicating a higher threshold than UFH.^3^ This range reflects the pharmacodynamics of enoxaparin and its suitability for venovenous ECMO settings.

#### ACT monitoring of UFH anticoagulation: Applications and limitations

Activated clotting time remains a choice in anticoagulation management during ECMO on UFH due to its simplicity and accessibility. The most commonly used ACT ranges from 140 to 210 s, with an average of 175.5 s and a median of 180 s. The latter is the most frequently reported target in clinical practice. Despite its widespread use, ACT has notable limitations that necessitate careful interpretation in critically ill patients.^8^, ^17^, ^22^, ^23^, ^24^

##### Limitations of ACT monitoring

ACT lacks standardization across devices, is influenced by hematocrit levels, hemodilution, and coagulopathy, and does not directly correlate with heparin activity.^31^, ^32^ ECMO circuit interactions, point-of-care inconsistencies, and environmental factors compound its variability. Additionally, ACT provides limited sensitivity at extreme heparin doses and fails to measure anti-Xa activity, making it a sole monitoring method less reliable. These limitations can lead to suboptimal anticoagulation management, with increased risks of bleeding or thrombotic complications.

##### Conclusion

While ACT monitoring is widely implemented, its limitations highlight the importance of combining it with complementary tests, such as anti-Xa activity or aPTT, to achieve accurate and safe anticoagulation management during ECMO.

#### UFH anticoagulation during ECMO using VEA

Many ECMO centers use VEA for anticoagulation monitoring, though approaches vary among institutions.^33^, ^34^ Limited data from published studies, primarily small prospective or retrospective observational research, indicate that results from different VEA instruments are not interchangeable.^25^, ^35^, ^36^ Moreover, there are significant differences in the coagulation profiles of patients on V-A and V-V ECMO, highlighting the need for VEA-based anticoagulation strategies to be tailored to both the specific VEA device and the ECMO modality.^37^ To date, only two studies, including one randomized clinical trial (RCT), have evaluated the feasibility of VEA-based anticoagulation protocols in ECMO patients. In the RCT, TEG 5000 was used to guide anticoagulation during V-V ECMO, targeting an R parameter of 16–24 min for individualized adjustments. The study found that TEG-guided management reduced bleeding complications compared to the aPTT-guided group, suggesting improved hemostatic balance without increased thrombotic risks.^18^ The second study, conducted by Colman et al., examined a combination of aPTT and TEG 5000-based protocols in V-A ECMO patients. Their protocol defined therapeutic anticoagulation as an aPTT of 60–80 s and a TEG-R time of 2–4 times the baseline. The results showed significantly lower mortality (33.3% vs. 56.9%, p = 0.01) and reduced retroperitoneal bleeding compared to aPTT alone.^38^ These findings suggest that TEG-based anticoagulation is safe and feasible in ECMO patients. However, further research is needed to validate these results and refine protocols.

#### Monitoring and supplementation of AT III during ECMO

Antithrombin III (AT III) plays a critical role in enhancing the efficacy of UFH during ECMO by serving as a natural coagulation inhibitor. UFH binds to AT III, significantly accelerating its ability to inactivate thrombin (factor IIa) and factor Xa, key enzymes in the coagulation cascade. This interaction amplifies the anticoagulant effect of UFH, helping to prevent thrombus formation within the ECMO circuit. Adequate AT III levels are, therefore, essential for optimal UFH activity. Studies have evaluated optimal AT III levels and thresholds requiring supplementation to reduce bleeding and thrombotic risks. Most studies defined normal AT III levels as 80–120% or 80–100%. Supplementation was recommended if AT III levels fell below 40%, 50%, or 70%, depending on the study.^17^, ^18^, ^24^, ^25^, ^30^ Maintaining AT III levels above 70% significantly reduced bleeding complications. Patients with low AT III levels often exhibited heparin resistance and required higher doses of UFH. Studies reported decreased bleeding complications with appropriate supplementation. Maintaining AT III levels within the 80–120% range is recommended for effective anticoagulation management during ECMO. Supplementation should be considered if levels fall below 50–70% to optimize heparin efficacy and minimize risks.^18^, ^24^, ^25^, ^30^

### Summary of anticoagulant agents for ECMO

For **UFH** bolus, the dose ranges from 50–100 IU/kg during ECMO cannulation; alternative protocols include fixed doses of 2500–5000 IU or 3000–5000 IU. Continuous Infusion Rates: 10.4–21.3 IU/kg/h, depending on monitoring parameters. Lower fixed-dose regimens include 12–15 IU/kg/h or 8000–12,000 IU/day based on patient weight.^8^, ^16^, ^17^, ^18^, ^19^, ^20^, ^23^, ^24^, ^25^, ^26^, ^27^, ^28^, ^30^, ^39^ For **enoxaparin,** a bolus dose of intravenous 0.5 mg/kg was administered before ECMO cannulation, followed by continuous administration, with anti-Xa target levels of 0.4–0.6 IU/mL.^3^ One study recommends a dosage of **argatroban** of 5–10 μg/kg/hour, with a target aPTT range of 45–60 s^4^ This dosing regimen aligns with standard clinical practice, where the initial infusion rate is typically set at 0.5–1.0 µg/kg/min and titrated to achieve an aPTT target of 1.5–2.0 times the baseline value.^2^ **Bivalirudin** bolus doses were not explicitly reported in the analyzed studies. At the same time, continuous infusion rates typically start at 0.025–0.15 mg/kg/h,^19^, ^28^, ^29^ with adjustments to 0.04–0.15 mg/kg/h for patients undergoing CRRT.^28^ **Prostaglandin E1 (PGE1)** is administered as a bolus dose of 50 IU/kg immediately after ECMO cannulation, followed by a continuous infusion of 5 ng/kg/min.^21^

#### Practical implications for anticoagulation management

**UFH** is the most commonly used anticoagulant, with flexible dosing strategies tailored to aPTT, anti-Xa, and ACT monitoring. It is also recommended for both VA and VV ECMO. **Enoxaparin** is a viable alternative to UFH, particularly in VV ECMO, with higher anti-Xa targets providing stable anticoagulation. **DTi** are especially suitable for patients with HIT or where precise anticoagulation is necessary. Lower initial doses can minimize bleeding risks while maintaining efficacy. **PGE1** is used adjunctively for its vasodilatory and antithrombotic properties, particularly in challenging ECMO cases. This comprehensive dosing overview highlights the need for individualized anticoagulation strategies to balance bleeding and thrombotic risks during ECMO. Further research is warranted to refine dosing protocols and validate their clinical efficacy across diverse patient populations.

## Advances in anticoagulation monitoring and therapy in ECMO

The choice of anticoagulant therapy and monitoring methods in ECMO support varies significantly across institutions. However, there is a clear trend toward developing sophisticated institutional guidelines to standardize anticoagulation therapy and its monitoring. Another observable trend is using the lowest possible anticoagulation doses to prevent thrombus formation while minimizing the risk of bleeding. Interestingly, global society guidelines and consensus statements recommend anticoagulant dosages and monitoring methods with reference ranges that have remained mostly unchanged for many years.^1^, ^2^, ^5^, ^6^, ^7^, ^12^, ^13^ However, scientific literature and ongoing studies indicate that routine clinical practice evolves in a different, more flexible direction. This is particularly evident in the trend toward lowering reference ranges for APTT, anti-Xa, and ACT, as well as the administration of significantly lower anticoagulation doses than those recommended by international societies. This shift can be reasonably explained by the fact that current studies do not yet provide sufficiently robust evidence to establish these practices as the standard of care. Consequently, while clinical practice adapts to emerging findings, formally incorporating these trends into global guidelines will likely require further high-quality evidence. A notable challenge in ECMO anticoagulation lies in the persistent reliance on ACT as a principal method for monitoring UFH therapy despite recommendations from international societies. Clinical practice increasingly acknowledges that ACT may not reliably reflect the anticoagulation effects of lower UFH doses commonly used in ECMO therapy, particularly when complicated by coagulopathy or organ failure. This has driven the search for novel, bedside-appropriate methods to provide reliable and actionable insights into anticoagulation status.

### Viscoelastic methods in the management of patients on ECMO

Over the past two decades, the TEG 5000 (Haemonetics Corporation) and ROTEM delta (Werfen) have been the primary VEA technologies, often referred to as "legacy devices." These systems require manual blood pipetting. To enhance precision, minimize human errors, and simplify assay performance, companies have developed newer generations of cartridge-based automated VEAs: TEG 6 s (Haemonetics), ROTEM sigma (Werfen), and Quantra (HemoSonics). Each utilizes a distinct methodology—resonance (TEG 6 s), traditional pin-and-cup (ROTEM sigma), and ultrasound-based technology (Quantra). Due to these methodological differences, the absolute values of viscoelastic parameters are not directly interchangeable across these devices. A detailed review of each assay, its limitations, and its capability to detect the effects of heparin have been previously published.^40^, ^41^ Viscoelastic testing methods, such as ROTEM and TEG, have emerged as promising tools in coagulation monitoring and targeted therapy.^8^, ^9^ These methods, already established for bedside diagnostics in coagulopathy management, offer significant potential to replace ACT in ECMO anticoagulation monitoring. For instance, the ROTEM Sigma system enables simultaneous assessment of four key parameters—EXTEM, FIBTEM, INTEM, and HEPTEM—using a single "all-in-one" cartridge. EXTEM and FIBTEM tests provide a rapid overview of secondary hemostasis, facilitating the identification of deficiencies in clotting factors, fibrinogen, or platelets. INTEM and HEPTEM tests are particularly relevant for UFH management. The clotting time (CT) INTEM/HEPTEM ratio (I/Hr) has been proposed as a potential marker for UFH efficacy, with values >1.25 indicating sufficient anticoagulation and serving as a trigger for protamine administration in cardiothoracic patients.^42^ This approach could be adapted to ECMO for effective UFH dosing and anticoagulation control. The mechanism behind this innovation is straightforward. UFH naturally prolongs the CT INTEM parameter, while HEPTEM neutralizes UFH's effects via heparinase, normalizing the CT. A similar effect, this time in terms of the difference between CT INTEM and HEPTEM, has been described with prophylactic administration of LMWH, which showed a strong correlation with the R parameter on TEG and, ultimately, with anti-Xa values.^43^ The correlation between I/Hr and established tests such as APTT, anti-Xa, and ACT requires further investigation. Still, early data suggest the potential for a simple, effective bedside method to manage ECMO anticoagulation.^43^, ^44^, ^45^, ^46^, ^47^ In addition to evaluating heparin, VEA can detect specific issues that patients may experience, such as excessive anticoagulation, underlying factor deficiencies, fibrinolysis, or disseminated intravascular coagulation. This capability is significant for guiding patient management, especially ECMO therapy patients. Determining which next-generation VEA most accurately reflects true hemostatic changes in ECMO patients remains a topic for future research.

### Antiagregation effects of ECMO on coagulation

ECMO therapy induces rapid primary hemostasis pathology, as evidenced by PFA-200 testing, which detects shear stress-induced thrombocytopathy and impaired platelet aggregation described by Garaj et al.^48^, ^49^ This phenomenon may justify the trend toward reduced systemic anticoagulation doses, as the combination of primary hemostasis dysfunction and low-dose anticoagulation exerts a synergistic effect that effectively prevents thrombus formation in the ECMO circuit and vascular system. This dual-effect anticoagulation combined with shear-stress-induced anti-aggregation could be particularly advantageous in specific scenarios, such as extracorporeal cardiopulmonary resuscitation (ECPR) during acute coronary syndrome requiring percutaneous coronary intervention (PCI) and stent implantation. In such cases, prophylactic doses of UFH, LMWH, or DTIs might suffice without the need for dual antiplatelet therapy, as platelet aggregation is already impaired by ECMO-induced shear stress.

## Future directions

Since early 2020, there has been a significant rise in patients with COVID-19-related acute respiratory distress syndrome (ARDS) requiring V-V ECMO. This surge has driven an exponential increase in ECMO centers and procedures worldwide. Over the past five years, 116,504 patients globally, including 83,301 in North America, have been treated with ECMO, but only 53% survived to discharge or transfer.^50^ Despite technological advancments, such as coated circuits, bleeding and thrombosis remain critical complications of ECMO.^51^, ^52^ Managing the delicate balance between thrombosis and bleeding through anticoagulation, flow rates, and fluid levels poses a significant challenge in ECMO patient care. The development and validation of advanced hemostasis monitoring techniques, such as VEA, hold great potential for improving anticoagulation management in ECMO therapy. These methods enable more precise anticoagulant dosing and provide a comprehensive understanding of coagulation dynamics, paving the way for safer and more individualized ECMO protocols. Furthermore, integrating machine learning into ECMO management offers substantial promise for enhancing patient outcomes. Machine learning can support more personalized and accurate care strategies^53^, ^54^ by better predicting the risks of hemorrhage and thrombosis. As evidence continues to grow, adopting these innovative approaches will likely to redefine the standard of care in ECMO therapy, contributing to improved patient outcomes and overall treatment efficacy.

*This review was written without receiving funding from any public, commercial, or non-profit organizations.*

## CRediT authorship contribution statement

**Jaromir Vajter:** Conceptualization, Methodology, Review of articles, Writing - original draft. **Oksana Volod:** Validation, Review of articles, Writing - review and editing.

## Declaration of Competing Interest

The authors, Jaromir Vajter and Oksana Volod, declare that they have no known competing financial interests or personal relationships that could have appeared to influence the work reported in this manuscript.
